# Integrating Evidence of the Traditional Chinese Medicine Collateral Disease Theory in Prevention and Treatment of Cardiovascular Continuum

**DOI:** 10.3389/fphar.2022.867521

**Published:** 2022-03-15

**Authors:** Iokfai Cheang, Shengen Liao, Qingqing Zhu, Gehui Ni, Cong Wei, Zhenhua Jia, Yiling Wu, Xinli Li

**Affiliations:** ^1^ Department of Cardiology, The First Affiliated Hospital With Nanjing Medical University, Nanjing, China; ^2^ National Key Laboratory of Collateral Disease Research and Innovative Chinese Medicine, Shijiazhuang, China; ^3^ Hebei Yiling Hospital, Key Disciplines of State Administration of TCM for Collateral Disease, Shijiazhuang, China

**Keywords:** cardiovascular disease, cardiovascular continuum, translational medicine, traditional Chinese medicine, set prescriptions

## Abstract

Cardiovascular disease has become a major public health problem. The concept of “cardiovascular continuum” refers to the continuous process from the risk factors that lead to arteriosclerosis, vulnerable plaque rupture, myocardial infarction, arrhythmia, heart failure, and death. These characteristics of etiology and progressive development coincide with the idea of “preventing disease” in traditional Chinese medicine (TCM), which corresponds to the process of systemic intervention. With the update of the understanding *via* translational medicine, this article reviews the current evidence of the TCM collateral disease theory set prescriptions in both mechanical and clinical aspects, which could lead to the development of new therapeutic strategies for prevention and treatment.

## Background

The cardiovascular continuum refers to a chain of events that begins from a host of cardiovascular risk factors and continues as a progressive pathogenic process leading to late complications, such as heart failure and myocardial infarction (Dzau and Braunwald, 1991). Despite the recent advancement in cardiovascular disease (CVD) management, population aging and social development continue to contribute to the growing economic and social burden (Glovaci et al., 2019).

Traditional Chinese medicine (TCM) has years of accumulated experience in disease management. TCM was often considered as a complementary and alternative approach to the primary and secondary prevention of CVDs (Xu et al., 2013). Although the efficacy and safety of TCM remain to be explored (Hao et al., 2017), the emerging evidence has shown their unique pharmacological effects of various active ingredients on the cardiovascular system and potential mechanisms on improving outcomes in patients with CVDs. Also, the generalization of the TCM has been restricted to the Chinese community due to the language limitations, the regionality of the origin, and the incomprehensible abstract theory. TCM needs to be further confirmed by modern medical theory and translates in the modern and uniform linguistic system in order to be widely recognized internationally.

The collateral disease theory is one of the major theoretical systems of the TCM (Ma Y. et al., 2016; Hao et al., 2017). While the main channel system has been emphasized in the past, collateral disease theory has been inexplicably neglected. The collateral disease theory has been emerging along with the development of TCM. In the aspect of collateral disease, diseases are caused by the blockage of meridian qi and blood, which is interestingly in line with the modern “cardiovascular continuum” fundamental mechanism (Wu, 2009).

Herein, the aim of this review was to introduce the research regarding these TCM set prescriptions in recent years, and to integrate the past evidence address of their possible therapeutic mechanism toward cardiovascular diseases.

## Prescriptions of Collateral Disease Theory in the Cardiovascular Continuum

**FIGURE 1:**
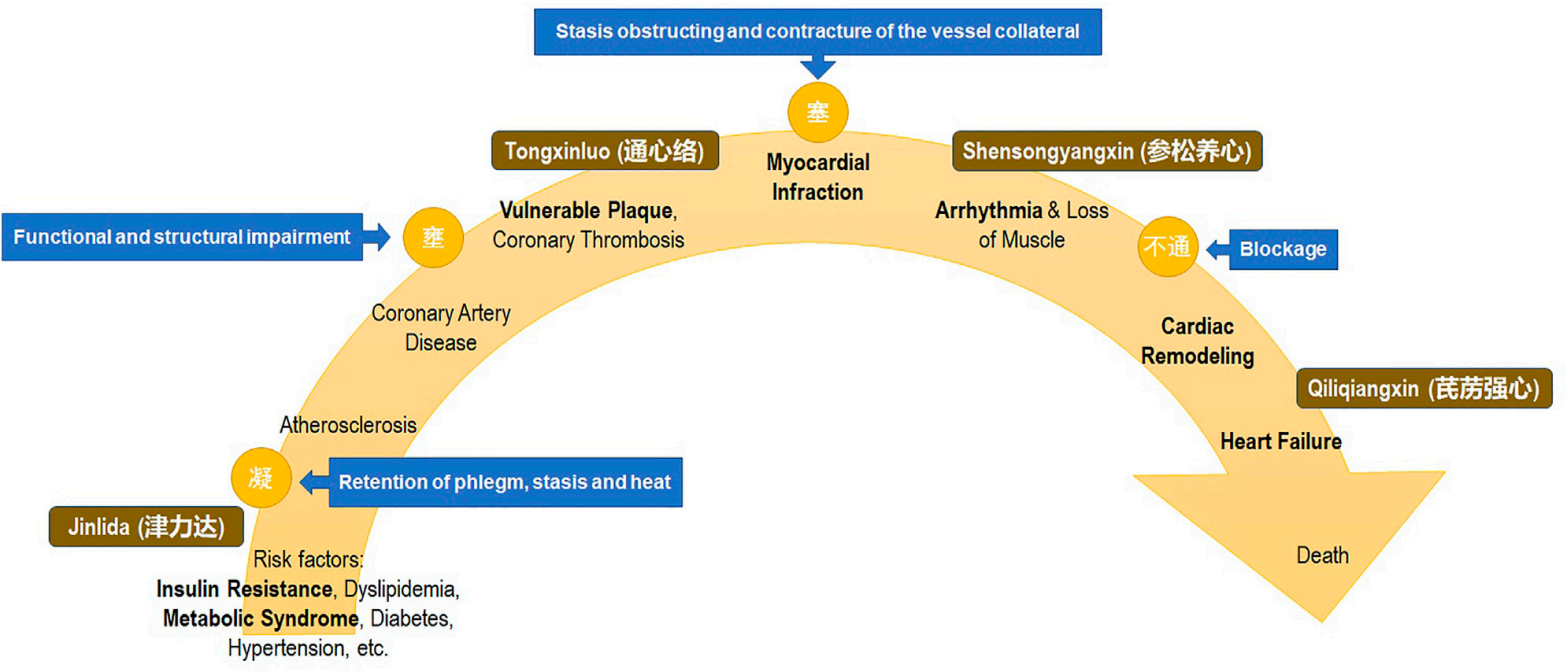
Cardiovascular continuum and intervention with traditional Chinese medicine set prescription of collateral disease theory.

Regarding these overlapped key nodes of the cardiovascular continuum, there are TCM set prescriptions based on collateral disease that could offer intervention targets at various continuum stages (Figure 1). For example, Jinlida granules (JLDG) for metabolic syndrome, Tongxinluo (TXL) for atherosclerotic plaque and myocardial protection, Shensongyangxin (SSYX) capsules for anti-arrhythmias *via* metabolic reconstruction, and Qiliqiangxin (QLQX) capsules for cardiac remodeling and heart failure have been recognized under the guidance of translational medicine (Table 1) (Bai et al., 2013; Wang et al., 2014; Zaki et al., 2014; Luan et al., 2015; Wang et al., 2015; Zhang et al., 2016; Fan et al., 2017; Shen et al., 2017; Zhao et al., 2017; Chen H. et al., 2018; Chen Y. et al., 2018; Gao et al., 2018; Wang et al., 2018; Zhang et al., 2018; Lyu et al., 2019; Tung et al., 2019; Zhou et al., 2019; Cheng W. et al., 2020; Li et al., 2020; Yang et al., 2020; Zhao et al., 2020; Jiang et al., 2021; Hao et al., 2022). These set prescriptions have been approved for by the Chinese National Medical Products Administration (NMPA), and broadly used in clinical practice in the Chinese community. However, the exact mechanism of these medicines on the modern evidence-based aspects was lacking in evidence-based translation to the modern medicine in the past.

**TABLE 1:** Components of the set prescriptions.

Set prescription	Species	Number of components	Representative herbs	Bioactive ingredients	Potential related mechanisms
Jinlida granules (JLDG)	Panax ginseng C.A. Meyer, Polygonatum kingianum Coll. et Hemsl, Atractylodes lancea (Thunb.) DC, *Sophora* flavescens Ait, Ophiopogon japonicas (L.f) Ker-Gawl, Rehmanniag lutinosa Libosch, Polygonum multiflorum Thunb, Cornus officinalis Sieb.et Zucc, Poriacocos (Schw.) Wolf, Eupatorium fortune Turcz, Coptis chinensis Franch., Anemarrhena asphodgfoides Bge, Epimedium brevicornu Maxim, Salvia miltiorrhiza Bge., Lycium chinense Mill., Pueraria lobata (Willd.) Ohwi, Litchi chinensis Sonn	17	Panax ginseng, Sophorae flavescentis Radix, Polygonati Rhizoma, Atractylodis Rhizoma	Ginsenosides (Rb1, Rc, Rb2), salvianic acid epimedin (B, C), Atractylodin, icariin	Metabolic regulation Wang et al. (2015); Wang et al. (2018); improve mitochondrial biogenesis Zhou et al. (2019); systemic anti-inflammation Lyu et al. (2019); Hao et al. (2022)
Tongxinluo (TXL) capsules	Panax ginseng C.A. Meyer, Paeonia lactiflora Pall., Ziziphus jujuba Mill. Var. spinosa (Bunge) Hu ex H.F.Chou, Santalum album L., Dalbergia odorifera T.C.Chen, Steleophaga plancyi (Boleny), Scolopendra subspinipes mutilans L. Koch, Hirudo nipponica Whitman, Cryptotympana pustulata Fabricius, Buthus martensii Karsch, Boswellia carteri, Borneolum syntheticum	12	Panax ginseng, Dalbergia odorifera, Boswellia carteri, Hirudo	Ginsenosides, Frankincense, Hirudinoidines	Inhibit ox-LDL-induced macrophage apoptosis Yifei Chen et al. (2018); alleviate ischemic/reperfusion-injury *via* activating PPAR-α and PI3K/Akt/eNOS/NO pathway Wang et al. (2014); Fan et al. (2017); anti-fibrosis and antioxidant effect Bai et al. (2013); Zaki et al. (2014)
Shensongyangxin (SSYX) capsules	Panax ginseng C.A. Meyer, Salvia miltiorrhiza Bge, Nardostachys jatamansi Dc., Cornus officinalis Sieb.et Zucc., Taxillus chinensis (DC.) Danser, Paeonia lactiflora Pall., Schisandra sphenanthera Rehd.et, Coptis chinensis Franch., Ophiopogon japonicas (Thunb.) Ker-Gawl., Polypodiodes chinensis, Eupolyphaga sinensis Walker, Ziziphus jujuba Mill. var. spinosa (Bunge) Hu ex H. F. Chou	12	Panax ginseng, Nardostachys jatamansi, Paeonia lactiflora , Coptis chinensis, Ophiopogon japonicus	Ginsenosides, Salvianolic acid B, Paeoniflorin, Berberine, Polysaccharide	Ion channel regulation effect Yang et al. (2020); Zhao et al. (2020); regulation of autonomic nerve ctivity Zhao et al. (2017); promoted angiogenesis Li et al. (2020); Jiang et al. (2021); anti-inflammation and remodeling Zhang et al. (2016); Hengwen Chen et al. (2018)
Qiliqiangxin (QLQX) capsules	*Astragalus* membranaceus (Fisch) Bge., Var. mongholicus (Bge.) Hsiao., Panax ginseng C. A. Mey., Aconitum carmichaeli Debx., Salvia miltiorrhiza Bge., Lepidium apetalum Willd., Alisma orientalis (Sam.) Juzep., Polygonatum odoratum (Mill.) Druce, Carthamus tinctorius L., Periploca sepium Bge., Cinnamomum cassia Presl, Citrus reticulata Blanco	11	*Astragalus* membranaceus, Aconitum carmichaeli, Salvia miltiorrhiza., Carthamus tinctorius, Cinnamomum cassia, Citri reticulatae Pericarpium	*Astragalus* polysaccharide, Diterpenoid Alkaloids, Salvianolic acid B, Hydroxysafflor yellow A, Cinnamomi ramulus, Nobiletin	Upregulation of PPARγ and PGC1α Shen et al. (2017); Gao et al. (2018); attenuate anoxia-induced injuries via NRG-1/ErbB-PI3K/Akt/mTOR pathway Regulating Energy Metabolism *via* HIF-1α and TNF-α/PGC-1α Zhang et al. (2016); Luan et al. (2015); Cheng W. et al. (2020); inhibit apoptotic response through IGF-I pathway Tung et al. (2019)

Therefore, researchers have made substantial efforts by clarifying the important scientific issues in the past decade. To identify the characteristics and targeted pathway by the modern scientific protocol, their mechanism and clinical effects have been partially translated to achieve a cross-integration of cardiovascular management (Tang and Huang, 2013; Hao et al., 2015). Although there are further questions that remained to be clarified, we believe that integrating the useful elements of TCM could expand the research field of CVDs and could also benefit the effective prevention and precise treatment.

## Translation in Modern Medicine

### Jinlida Granules in Regulating Metabolism

Systemic risk factors such as obesity, diabetes, and dyslipidemia often exist in the pathophysiological process of atherosclerotic cardiovascular disease (ASCVD), which could lead to adverse outcomes. A large number of clinical and experimental studies have confirmed that energy imbalance caused by excessive energy intake and reduced consumption is the main cause of obesity and glucose and lipid metabolism disorders. Furthermore, inflammation is associated with increased oxidative stress, platelet activation, vascular endothelial dysfunction, and other metabolic CVD which are closely related to these pathophysiological changes (Kolwicz et al., 2013; Schmidt, 2019; Wang et al., 2019; Glatz et al., 2020).

Treatment targeting metabolic aspects (such as SGLT-2 inhibitors) may significantly reduce the risk of atherosclerotic cardiovascular and cerebrovascular diseases (Ren et al., 2010; Hou et al., 2020). The balance of intake and consumption of energy substances is the basis for maintaining the normal physiological functions of the body. Insulin resistance as the core mechanism of metabolic syndrome is often associated with abnormal blood glucose regulation (Rochlani et al., 2017). Previous studies showed that Jinlida granules (JLDG) might be beneficial *via* their metabolic regulation effects by protecting islet β-cell (Shi et al., 2013), and play a role in various pathways in anti-metabolic disorders. JLDG could reduce insulin resistance by regulating the lipid metabolism (Wang et al., 2015), promoting skeletal muscle gene and protein expression (Zang et al., 2015). JLDG also improves metabolic disorders associated with the activation of brown adipose tissue (BAT) thermogenesis *via* enhancement of mitochondrial biogenesis and fatty acid oxidation metabolism (Zhang H. et al., 2019).

Furthermore, JLDG demonstrated the effect of anti-oxidative stress (Liu et al., 2015) and regulating hormones related to blood glucose (Pang et al., 2014). Systemic anti-inflammatory protective effect of JLDG also reflected in improving NAFLD by antagonizing hepatocyte pyroptosis in the high-fat-diet-induced liver injury mice model (Hao et al., 2022).

Clinical trials have also shown the efficacy of JLDG in diabetes treatment (Lian et al., 2015; Shi et al., 2016; Tian et al., 2018; Pan et al., 2021). Furthermore, a meta-analysis (Lian et al., 2019) that included clinical studies in the past decade showed the treatment with JLDG provided clinically and statistically significant reductions in fasting plasma glucose, 2-h postprandial plasma glucose, and the glycated hemoglobin (HbA1c) level in patients with type 2 diabetes mellitus.

These results suggest that JLDG might be a complementary therapeutic agent or even treatment regimen for metabolic syndrome and diabetes. The exploration in the mechanism of the ingredients and active monomers would contribute to recognizing the metabolic properties and its therapeutic effects in CVD.

### Tongxinluo in Atherosclerosis

Atherosclerosis is associated with chronic and progressive inflammation (Zhu et al., 2018). Inflammation damage to endothelial cells plays an important role in the initiation and progression of atherosclerotic plaque. Factors such as cells and cytokines are involved in this process, namely, macrophages, lymphocytes, dendritic cells, endothelial cells, vascular smooth muscle cells, interleukin, adhesion molecules, and tumor necrosis factor (TNF-α) are involved in the process. At the same time, pathological changes including abnormal lipid metabolism, hemorheological changes, oxidative stress, intimal hyperplasia, and adventitia nourish angiogenesis interrelatedly promote atherosclerosis (Gimbrone and García-Cardeña, 2016; Poznyak et al., 2020).

As a TCM set prescription, studies have found that Tongxinluo (TXL) can regulate the lipid metabolism and anti-atherosclerosis, and improve atherosclerotic plaque stability through various pathophysiological pathways. One major pathway includes the alleviation of plaque inflammation by inhibiting inflammation-induced neovascularization in the plaque *via* inhibition of the NLRP3 pathway to stabilize the atherosclerotic plaque (Ma et al., 2015; Wang et al., 2021). TXL could regulate the lipid metabolism (Ma L. et al., 2016; Zhou et al., 2016; Chen et al., 2021) which strongly correlate to the composition of the intestinal flora and intestinal metabolites associated with the stability of plaque by promoting the adenosine triphosphate-binding cassette transporter A1 (ABCA1) (Li Y. et al., 2021; Huang et al., 2021). Furthermore, TXL protected the endothelial barrier integrity in reperfused diabetic rats’ hearts *via* peroxisome proliferator-activated receptors—alpha (PPARα) pathway independent of the blood glucose level (Bai et al., 2013). TXL has effects. The comprehensive mechanisms of TXL on inhibiting atherosclerosis development and stabilizing plaque might also involve cell physical function, hormone secretion, protein binding, and immune response process (Ma et al., 2019). In a clinical observation study (CAPITAL study), in addition to conventional treatments in patients with subclinical AS, treatment with the TXL group showed a significant delay in the progress of average IMT, plaque area, and carotid vascular remodeling without additional adverse drug safety outcomes (Zhang M. et al., 2019).

On the other hand, myocardial no-reflow is associated with microvascular endothelial damage, microthrombosis, microvascular spasm and injury, myocardial ischemia/reperfusion (I/R) injury, and microvessel dysfunction. In addition, these pathological changes of inflammation, oxidative stress, calcium overload, and mitochondrial dysfunction were shown to be persistent after reperfusion and suggested that the recovery is a continuous dynamic process (Kang et al., 2010).

Studies have shown that pre-administration of TXL could reduce the myocardial no-reflow area and myocardial infarction area in animal models (Li et al., 2006; Qian et al., 2007; Cheng et al., 2009). The specific mechanism may be related to the downregulation of miR-128-3p and promotion of p70s6k1 protein expression (Chen et al., 2017), the activation of the PKA-eNOS pathway (Li et al., 2010), and the activation of the MEK/ERK pathway, vascular endothelial growth factor (VEGF), angioprotein-like protein-4, granulocyte colony-stimulating factor (G-CSF) (Li X. L. et al., 2013), AMP-activated protein kinase (AMPK) (Li et al., 2017), *etc*. Also, TXL treatment significantly inhibited macrophage apoptosis by enhancing macrophage autophagy by increasing Beclin-1 expression and improving Bcl-2–Beclin-1 complex dissociation (Chen Y. et al., 2018). Recent studies have further demonstrated TXL could reduce myocardial/endothelial cell apoptosis and necrosis due to both hypoxia and reperfusion *via* upregulating the expression level of lincROR, downregulating miRNA145-5p, activating the p70s6k1/eNOS signaling pathway (Chen et al., 2020), and downregulating the expression of MMP family proteins in macrophages (Ma et al., 2019).

In a clinical aspect, the ENLEAT study (Zhang et al., 2010) also showed that TXL can promote myocardial reperfusion, significantly reduce the incidence of coronary no-reflow after reperfusion, reduce the area of myocardial infarction, and improve the cardiac systolic function for STEMI patients in addition to conventional medicine therapy. An ongoing randomized control trial CTS-AMI study regarding the efficacy and safety of TXL will provide more comprehensive evidence for the patients with coronary artery disease (Xu et al., 2020).

### Shensongyangxing in Metabolic Reconstruction

However, metabolic syndrome (MS) is closely related to an increased morbidity of arrhythmia in various pathophysiological aspects (Kumar and Gehi, 2012; Gawałko et al., 2021; Joseph et al., 2021; Pool et al., 2021). A large cohort study showed that metabolic factors including obesity, hypertension, low LDL-C level, and impaired fasting blood glucose level are closely related to new-onset atrial fibrillation (AF) (Watanabe et al., 2008). Metabolic syndrome is an independent predictor of AF associated with the left atrial low voltage zone (Dinov et al., 2014). Furthermore, MS is also associated with the increased risk of ventricular arrhythmia (VA) (Fernández-Sada et al., 2017; Liptak et al., 2017) and was reported to contribute to a higher recurrence rate of outflow tract VA after catheter ablation (Sardu et al., 2014). However, the underlying role of MS in the occurrence and development of such arrhythmia remains unclear. Theories have been raised that it might be related to the oxidative stress, inflammation, myocardial fibrosis, and ferroportin pathway along with the process of cardiovascular continuum.

Shensongyangxing (SSYX) has an anti-arrhythmic effect. There is research interpreting its possible mechanism of the effectiveness. Studies have shown that SSYX can inhibit AMPK phosphorylation and PGC-1α activity, thereby improving myocardial energy metabolism and improving Ang-II-induced primary cardiomyocyte hypertrophy (Liu et al., 2018). Another study showed that SSYX reduces the occurrence of AF after myocardial infarction by inhibiting atrial fibrosis (Ma et al., 2018). At the same time, SSYX has shown to be a multichannel blocker with measurable modulation effects on various ion channels, such as L-type calcium channel and transient outward potassium (Ito), resulting in an overall prolongation of the action potential (Yang et al., 2020; Zhao et al., 2020). SSYX could inhibit IK1 and Ito currents by prolonging the duration of action potentials, reversing calcium overload, and inhibiting the occurrence of ventricular arrhythmias after ischemia (Zhao et al., 2016). In a metabolic aspect, animal research showed that SSYX can reverse and alleviate the metabolic and arrhythmic pathological changes such as impaired glucose tolerance, enlarged atria, atrial fibrosis, atrial inflammation, and oxidative stress; shortened effective refractory period; and prolonged action potential duration induced by metabolic syndrome (Zaki et al., 2014; Chen Y. et al., 2018; Yang et al., 2020).

Previous multicenter clinical studies showed that SSYX capsule has a significant effect on the treatment of heart failure (HF) complicated by ventricular arrhythmia (Liu et al., 2014; Wang X. et al., 2017; Cao et al., 2021). SSYX capsules have also been demonstrated to be effective in the treatment of paroxysmal AF (Jiang et al., 2022). In cohorts without HF, studies have demonstrated the effectiveness of SSYX for the concomitant treatment of frequent premature ventricular contractions and sinus bradycardia (SB), and alleviation of the related symptoms (Zou et al., 2011; Zhang F. et al., 2019).

### Qiliqiangxin in Cardiac Remodeling

As an end stage in the development of various heart diseases, the morbidity and mortality of heart failure (HF) gradually increase due to population aging. Although some treatment progress has been made in the past decade, HF is still a major problem that seriously threatens human life and quality of life (Tomasoni et al., 2019). The basic mechanism of the occurrence and development in heart failure includes pathological cardiomyocyte hypertrophy with embryonic gene re-expression, cardiomyocyte apoptosis and necrosis, and excessive deposition or degradation of cardiomyocyte extracellular matrix (Gyöngyösi et al., 2017). The activation of peroxisome proliferator-activated receptors (gamma (PPARγ)) and its coactivator-1α (PGC-1α) play key roles in the amelioration of cardiac hypertrophy and dysfunction (Li et al., 2010). PPARγ also plays an important role in cardiac metabolism remodeling and the observed attenuation of cardiac fibrosis, inflammation, and ROS production (Wang et al., 2016; Penas et al., 2020).

Qiliqiangxin (QLQX) capsule is a compound preparation derived from TCM pathology which is in line with the modern prospective of neuroendocrine activation and ventricular remodeling in HF. QLQX can improve the heart function of HF rats and increase the excretion of water by reducing the expression of AQP2 in the kidneys (Cui et al., 2015). It can also reduce the expression of Ang II and periostin protein in HF rats after myocardial infarction (Zhou et al., 2015; Li F. et al., 2021), while the immunomodulatory effects of anti-inflammatory factors might be one of the immunopharmacological mechanisms of QLQX to improve the heart function of AMI rats. These previous cells and animal studies regarding the effect of QLQX in various models have shown the protective effects on inhibiting pathological cardiac remodeling, mechanistically *via* activation of PPARγ (He et al., 2013; Pan et al., 2014; Wang H. et al., 2017), and the protective effect of QLQX in the heart might also be attributed, but not limited, to the blood glucose reduction effect (Wu et al., 2021).

Further analysis of the QLQX component demonstrated that citri reticulatae Pericarpium (CRP) inhibits pathological cardiac hypertrophy (Cheng H. et al., 2020). CRP protected against pathological cardiac hypertrophy is induced by Ang II stimulation in neonatal rat cardiomyocytes (Ni et al., 2020), while the active monomer nobiletin isolated from CRP might be the major factor in attenuating the adverse cardiac remodeling via anti-inflammatory (Bunbupha et al., 2020) and antiapoptotic functions (Amarsanaa et al., 2021) similarly *via* activating PPARγ and PGC1α (Zhou et al., 2021). However, studies to explore the mechanism of QLQX for its protective effects on cardiac pathological hypertrophy, and the interaction of the component are still needed.

Regarding the clinical efficacy aspect, a multicenter randomized controlled clinical study (Li X. et al., 2013) showed that QLQX can significantly reduce the secondary endpoint of HF patients in addition to the standard HF treatment. The study assessing the prognosis on the hard endpoint-cardiovascular mortality and HF rehospitalization to further clarify the clinical efficacy and safety of QLQX is expected for further evidence-based confirmation (Yao et al., 2020).

## Conclusion

Although the findings should be interpreted with caution because the studies might only represent a limited aspect of the set prescriptions, the exploration of these TCM set prescriptions on cardiac metabolism pathways and the relevance of this knowledge for current clinical practice may have utility for the future treatment of patients with cardiovascular diseases. To be noted, we should also acknowledge the potential reactions caused by drug/component interactions when combining traditional Chinese medicine and modern medicine. We believe integrating the evidence of TCM could provide sufficient elucidation of the mechanism to the clinical effect and complement the current management approaches.

## Abbreviations

ABCA1, adenosine triphosphate-binding cassette transporter A1; AMPK, AMP-activated protein kinase; ASCVD, atherosclerotic cardiovascular disease; AF, atrial fibrillation; BAT, brown adipose tissue; CVD, cardiovascular disease; CRP, citri reticulatae Pericarpium; HBA1c, glycated hemoglobin; G-CSF, granulocyte colony stimulating factor; HF, heart failure; I/R, ischemia/reperfusion; JLDG, Jinlida granules; NMPA, Chinese National Medical Products Administration; MS, metabolic syndrome; PPARγ, peroxisome proliferator-activated receptors gamma; PCG-1α, PPAR gamma coactivator-1α; QLQX, Qiliqiangxin; RXR, retinoic acid X receptor; SSYX, Shensongyangxin; SGLT-2, sodium-glucose cotransporter-2; TXL, Tongxinluo; TCM, traditional Chinese medicine; VEGF, vascular endothelial growth factor; VA, ventricular arrhythmia.
